# Interaction between bradykinin and urodilatin - a possible mechanism of clinical relevance

**DOI:** 10.1186/1471-2210-11-S1-P21

**Published:** 2011-08-01

**Authors:** Marina Dobrivojević, Aleksandra Sinđić, Bojana Nikitović, Bayram Edemir, Eberhard Schlatter, Wolf-Georg Forssmann, Jochen R Hirsch

**Affiliations:** 1 Dept. Physiology, School of Medicine, Croatian Institute for Brain Research, University of Zagreb, Croatia, Rupublic of; 2 Universitätsklinikum Münster, Experimentelle Nephrologie, Münster, Germany; 3 Cardiopep Pharma GmbH, Hannover, Germany

## Background

Bradykinin (BK) plays a significant role in pathophysiology of different diseases from angioedema to brain stroke and heart attack by inducing vasodilatation and increasing capillary permeability. We investigate the potential effects of natriuretic peptides on BK signaling by measuring membrane potential (*V*_m_) of HEK293 cells using the whole cell patch clamp technique. HEK293-cells are an excellent model since they display the natriuretic peptide receptor type A as well as both BK receptors (BR1 and BR2).

## Results

**Figure 1:**
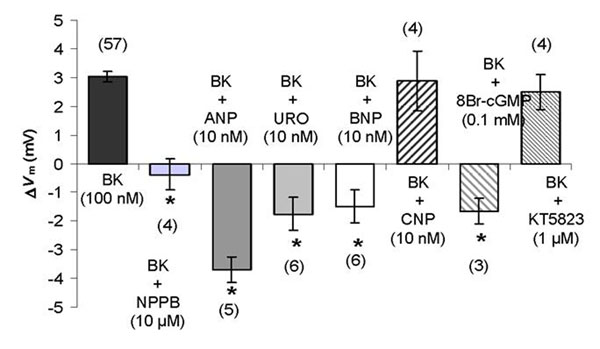
Effects of natriuretic peptides and cGMP signaling pathway on bradykinin (BK) action. BK – bradykinin; NPPB – inhibitor of Ca^2+^-dependent Cl^-^ channels; ANP – atrial natriuretic peptide; URO – urodilatin; BNP – brain natriuretic peptide; CNP – C-type natiuretic peptide; KT5823 – inhibitor protein kinase G. Number of experiments are given in brackets. * p<0.05 compared to depolarizations caused by bradykinin alone

Starting *V*_m_ was -53.5 ± 1.1 mV, n = 184. To detect viability of the cells hyperkalemic conditions were used (changing K^+^ from 3.6 mM to 18.6 mM) which depolarized HEK293 cells by 9.7 ± 0.4 mV, n = 182. BK (100 nM) depolarized HEK293 cells by Δ*V*_m_ = 3.0 ± 0.2 mV, n=57 (figure 1). Our preliminary results show that the BK induced depolarization by activating bradykinin receptor type 2 (BR2) and further down the signaling pathway activation of Ca^2+^-dependent Cl^-^ channels (Figure 1).

Natriuretic peptides display different effects on the BK-induced depolarization due to activation of Ca^2+^-dependent Cl^-^-channels. While ANP, urodilatin, BNP (all acting though guanylate cyclase A) and 8-Br cGMP inhibited the BK-induced depolarization, CNP (acting through guanylate cyclase B) failed to do so (Figure 1). Effects of urodilatin on BK-induced deoplarization could be reversed by inhibiting protein kinase G (using the specific inhibitor KT5823) suggesting an inhibitory role of natriuretic peptides via GC-A, cGMP and PKG in BK signaling.

## Conclusion

From our results we could speculate that natriuretic peptides might display beneficial effects in different pathological conditions caused by BK.

